# Case of Monstrosity

**Published:** 1852-07

**Authors:** M. Miles

**Affiliations:** Mich.


ART. VI.—*Case of Monstrosity.* By M. MILES, M.D., of Mich.

ON the 29th of April last I was called to visit Mrs. S——, in labor with her third child. I arrived at about 10 o'clock, A. M., and on making an examination found the left foot of the child presenting and protruding from the external parts. About nine inches of the cord was prolapsed, together with a firm, roundish, flattened body which felt like liver, although I at first mistook it for the placenta, as I could trace the cord to its edge, where it seemed to terminate.

I was informed that the patient had been in this situation about ten hours, although the pains were regular and active. Her pelvis was large and well formed and her previous labors had been natural. On a more minute examination I found the right leg of the child was flexed upon itself and wedged across the brim of the pelvis, thus preventing the hips from entering the superior strait. As pulsation had ceased in the cord, I made no attempt to return it, but introduced my hand and brought down the right leg, when delivery immediately took place.

The head and limbs of the child were well formed, while the body presented many peculiarities. The distance between the seventh cervical vertebra and the sacrum was about one-and-a-half inches, so that the hips appeared to be attached immediately to the neck. The thorax appeared large and well formed when seen in front, but on a side view it was nearly covered by the hips. The liver and intestines were external to the abdominal cavity; the convex surface of the liver was external, while the upper and a part of the lateral edges were attached to, and continuous with, the integument, thus assisting in forming the abdominal walls The intestines were suspended from the inferior edge of the liver, and just beneath them was a small opening into the abdomen.

The cause of the deformity was very satisfactorily accounted for by the "old women" who were present, as the patient when in the second month of pregnancy was chased across the fields by a bear.

